# Spatial and spectral coherence in propagating high-intensity twin beams

**DOI:** 10.1038/srep14365

**Published:** 2015-09-25

**Authors:** Ondřej Haderka, Radek Machulka, Jan Peřina, Alessia Allevi, Maria Bondani

**Affiliations:** 1 RCPTM, Joint Laboratory of Optics of Palacký University and Inst. Phys. AS CR, 17. listopadu 12, 77146 Olomouc, Czech Republic; 2 Institute of Physics AS CR, Joint Laboratory of Optics, 17. listopadu 50a, 77146 Olomouc, Czech Republic; 3 Dipartimento di Scienza e Alta Tecnologia, Università degli Studi dell’Insubria, Via Valleggio 11, 22100 Como, Italy; 4 CNISM UdR Como, via Valleggio 11, 22100 Como, Italy; 5 Istituto di Fotonica e Nanotecnologie, CNR, Via Valleggio 11, 22100 Como, Italy

## Abstract

Spatial and spectral coherence of high-intensity twin-beam states propagating from the near-field to the far-field configurations is experimentally investigated by measuring intensity auto- and cross-correlation functions. The experimental setup includes a moving crystal and an iCCD camera placed at the output plane of an imaging spectrometer. Evolution from the tight near-field spatial position cross-correlations to the far-field momentum cross-correlations, accompanied by changeless spectral cross-correlations, is observed. Intensity autocorrelation functions and beam profiles are also monitored as they provide the number of degrees of freedom constituting the down-converted beams. The strength of intensity cross-correlations as an alternative quantity for the determination of the number of degrees of freedom is also measured. The relation between the beam coherence and the number of degrees of freedom is discussed.

Recently, quite a lot of attention has been devoted to spatial correlations in photon-pair states generated by parametric down-conversion (PDC), serving both for tests of quantum mechanics and for applications in the area of quantum information processing^1^. Special attention has been paid to the propagation of the photon-pair states and the accompanying evolution of their entanglement. To this aim, a theoretical framework employing the concept of fractional Fourier transform has been elaborated and tested^2^,^3^ to describe the photon-pair state propagation in the simplest and the most intuitive way.

Earlier works on spatial correlations in PDC were performed by scanning point-like detectors^3^,^4^,^5^,^6^, while more recent approaches employ single-photon-sensitive iCCD^7^,^8^ or EMCCD cameras^9^,^10^. In fact, such cameras make it possible to get a more complete picture of the highly-dimensional Hilbert space of spatial PDC correlations at the single-photon level^11^ as they combine the advantages of single-photon detectors and the spatial resolution of CCD cameras, which in the past were exploited to investigate the high-gain PDC process only^12^.

Most investigations of spatial correlations to date have been performed either in the near-field or in the far-field configurations^4^,^5^,^7^, as well as in both^9^. In addition, attention has been devoted also to the transient area between the two extremes^3^,^6^,^13^,^14^. While in the near-field position intensity cross-correlations (XC) have been confirmed in^9^, momentum anti-correlations have been observed in the far-field^7^,^9^, as a result of the phase-matching conditions. In the transient regime, XC get blurred and, at a certain position, they cannot be observed at all^13^. At this position, entanglement is entirely transferred to the phase of the two-photon amplitude^13^, thus becoming hidden to intensity observations. The evolution of XC from near field to far field has been experimentally observed at the single-photon level^6^ and the Fedorov ratio^15^ has been determined. In the experiment reported in^6^, the Fedorov ratio equal to one has been measured at the position where XC were spread over the whole PDC beam.

Finally, we mention that also investigations of the far-field spectral correlations depending on pump-field parameters (power) have been performed by using imaging spectrometers combined with EMCCD cameras^16^. Nevertheless, their behavior in the transition from near field to far field has not been experimentally addressed yet.

We note that nearly all the above-mentioned investigations have been carried out in the single-photon regime, in which a well established theory based on the biphoton function exists. Here we are interested in investigating how those results compare to the analogous quantities in the high intensity regime. To this aim, in this paper, we experimentally demonstrate the physical mechanism ruling the behavior of spatial and spectral autocorrelations (AC) and cross-correlations in high-intensity PDC in the transition from the near field to the far field. To do this, we built a fixed optical system to image an object plane (see Fig. 1) to the plane of the vertical slit of an imaging spectrometer. The nonlinear crystal can be translated in the opposite direction with respect to the spectrometer, so that the object plane of the optical system is made to coincide with the output face of the crystal itself (near field, *z* = 24 mm), with the far-field-like configuration (*z* = 0 mm) and with all the planes in between. This system allows us to measure both spatial and spectral intensity correlations simultaneously, thus providing the overall picture of the evolving twin beam. The consistency of the experimental data is ensured by monitoring the spectral correlations even at the positions where the spatial XC get blurred or even lost. This experimental configuration allows us to observe a clear transition from position correlations in the near field to momentum anti-correlations in the far field monitoring the level of blurring of both types of XC. Finally, the size of spatial and spectral intensity cross-correlations provides the number of degrees of freedom of the twin beam. Moreover, the maximum heights of intensity XC peaks can be used to estimate the number of degrees of freedom in the field. The numbers of degrees of freedom are also obtained from AC measurements which completes the picture of the evolving twin beam.

## Results

**Figure 1:**
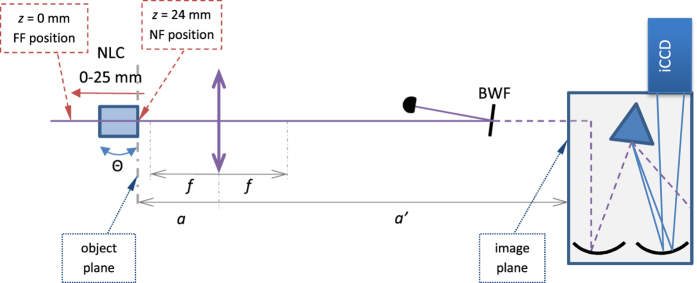
Sketch of the experimental setup. The nonlinear crystal is placed on a roto-translation stage. In the near-field position (NF, *z* = 24 mm), the lens with focal length *f* and the imaging spectrometer are placed (distances *a*, *a*′) so that the output face of the crystal is imaged to the plane of the input slit of the spectrometer. Without changing *a*, *a*′, the crystal is moved in the range 0 to 25 mm so that different propagation planes of PDC are always imaged to the input slit of the spectrometer. The bandwidth filter (BWF) deflects most of the pumping beam power. More details are given in Section “Methods”.

**Figure 2:**
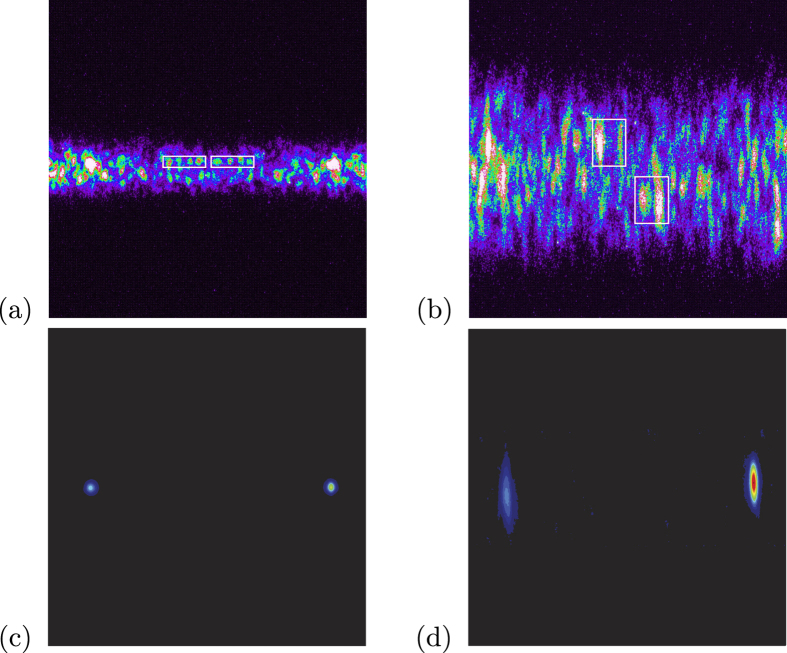
Typical camera images obtained in the near-field (**a**) (left, *z* = 24 mm) and far-field (**b**) configurations (right, *z* = 0 mm). White rectangles remark examples of correlated structures to illustrate the mirror symmetry (**a**) or the inverted-mirror symmetry (**b**). In panels (**c**,**d**) two examples of processed images, showing the AC and XC peaks, are displayed in the near-field and far-field configurations, respectively. Note that, as in both cases the reference point was chosen in the right-hand portion of the image, the AC peak is on the right and the XC peak is on the left. The horizontal axis spans the spectrum and the vertical axis corresponds to the radial spatial coordinate of the PDC cone.

In the experiment sketched in Fig. 1 (for details, see Section “Methods” below) the third harmonics of a picosecond-pulsed Nd:YLF laser was used to pump a type-I BBO crystal in a nearly-collinear interaction geometry. The BBO crystal was mounted on a roto-translation stage, whose movement allowed us to observe the transition from the near-field to the far-field configurations (see above). As anticipated, a fixed optical system was used to image different planes behind the crystal on the input slit of an imaging spectrometer, whose output was monitored by an iCCD detector. Typical images obtained with the camera in the experiment are shown in Fig. 2(a,b). In the pictures, we can observe the single-shot speckle-like random patterns appearing due to partial coherence established in the PDC process. In the far field [Fig. 2(b)] the beam is wider than in the near field [Fig. 2(a)] due to the divergence of the PDC cone. Upon careful inspection of the positions of the bright speckles one can recognize a mirror symmetry along the center of the images [(a), near field] or inverted mirror symmetry [(b), far field].

**Figure 3:**
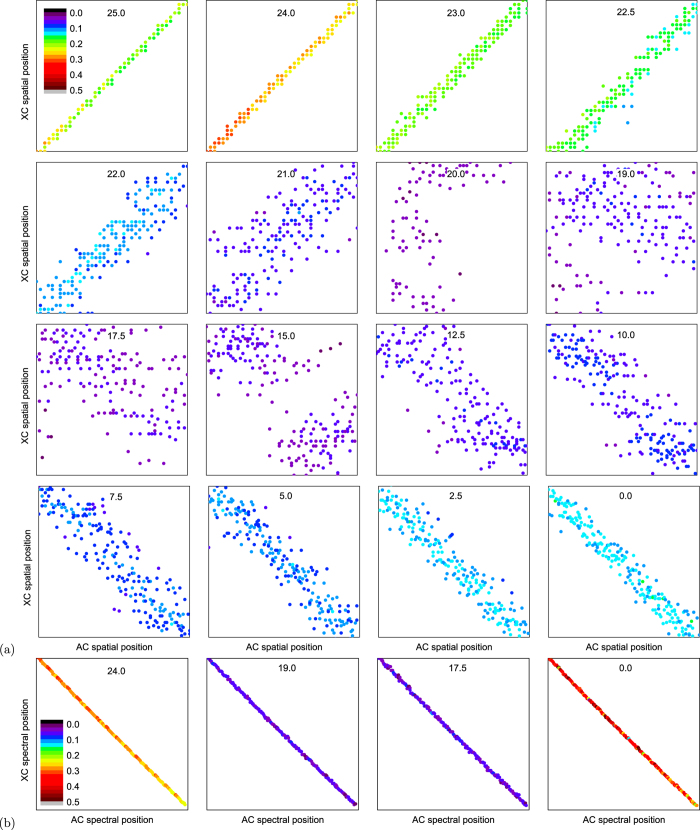
Spatial (**a**) and spectral (**b**) positions of the AC and XC peaks drawn in a 2D plot for different *z*-axis positions (the label at the top of each panel shows the position in mm). Each dot represents a spatial (**a**) (i.e., vertical, referring to Fig. 2) or spectral (**b**) (i.e., horizontal, referring to Fig. 2) position of the XC peak plotted against the position of the AC peak. Each panel in (**a**) is normalized to the size of the corresponding PDC beam FWHM to scale it to the divergence of the beam. The dots are color-coded (see the inset of the first panels) expressing the magnitude of the XC peak. The panels in (**b**) show the whole registered bandwidth (683.4 nm to 712.5 nm) in both coordinates.

We performed a scan of the spatial and spectral correlations as functions of the *z*-axis position. For each *z*-axis position, 100 points for each image were taken and processed to arrive at intensity AC and XC functions, *C*(*x*, *y*) (for details, see section “Methods” below). Typical examples taken in the near- and far-field configurations are shown in Fig. 2(c,d). Peak values, as well as peak widths, were identified for characterizing both correlation functions. We observe correlations in the positions of the intensity AC and XC peaks (see Fig. 3), which change their character as we move the crystal along the *z*-axis. In Fig. 3(a) we show spatial positions (vertical coordinate in Fig. 2) of the XC peaks against spatial positions of the corresponding AC peaks. Note that, to take into account the different divergence of the PDC beam at the transition from the near-field to the far-field configurations, in each panel the real positions on the camera were normalized to the size (FWHM) of the corresponding PDC beam. We note a sharp diagonal around the position *z* = 24 mm, which corresponds to the near field. The location of the near field at this position is also confirmed by the direct observation of the crystal output face at the plane of the input slit of the spectrometer when the spectrometer is switched to the imaging mode. Moving the crystal away from the spectrometer leads to blurring of the diagonal. Around position *z* = 20 mm, the diagonal is completely lost, so there is no spatial correlation in the positions of the peaks. Moving the crystal even further to lower *z* values, anti-diagonal character of the correlations (anti-correlation) is gradually established. Towards position *z* = 0 mm, the anti-diagonal becomes sharper, thus indicating the approach to the far-field momentum correlations. We are not exactly in the far-field configuration reached at *z* = −∞, but the far-field character of the correlations is clearly established at a distance exceeding 1 cm from the crystal.

In the transient area (between *z* = 20 mm and *z* = 15 mm), the cross-correlations in the PDC field are nearly lost only in the space. On the contrary, spectral cross-correlations (horizontal coordinate in Fig. 2) remain unchanged, as documented in Fig. 3(b). Here the tight anti-correlations emerging from the energy conservation are preserved at all *z* positions.

**Figure 4:**
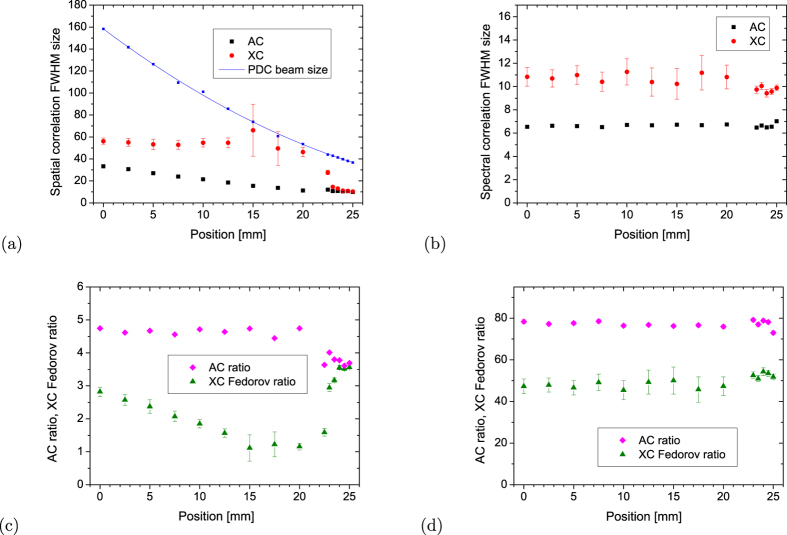
Spatial (**a**) and spectral (**b**) intensity AC (black, squares) and XC (red, circles) peak widths (FWHM) as functions of the *z*-axis position. Blue solid curve in (**a**) shows PDC spatial beam width (FWHM) at the input slit of the spectrometer. Vertical axes in (**a**) and (**b**) are expressed in binned-superpixel units (each superpixel corresponds to 2 × 2 physical pixels of the camera, i.e., the side of the superpixels is 26 *μ*m). In (**b**), a binned superpixel corresponds to 0.057 nm of spectral width. Spatial (**c**) and spectral (**d**) XC Fedorov ratio (green, triangles) and AC ratio (pink, diamonds) are plotted.

In addition to the position of the peaks, we also measured the AC and XC peak widths as a function of the *z*–axis position (see Fig. 4). We note that the near-field configuration corresponds to the right edge of the plots. In the near-field configuration, the AC and XC functions have nearly identical spatial widths. Moving towards the far-field (*z* = 0) the spatial AC width exhibits a mild growth, while the spatial XC width grows rapidly up to the PDC beam size. The PDC beam size was determined from the vertical, i.e. spatial, extent of the beam as obtained from a large number of accumulated images like those shown in Fig. 2(a,b) at each *z*-position. Note that the increase of spatial XC width occurs at the distances at which we find the completely blurred spatial diagonals plotted in Fig. 3(a). Going further towards the far field, momentum anti-correlations get established [see Fig. 4(a)] resulting in a gradual decrease of the spatial XC width.

The behavior of spatial XC widths can be used to investigate the XC Fedorov ratio, determined as the ratio of the width of the whole PDC beam to the XC width [see Fig. 4(c)]^6^,^13^. While the spatial XC Fedorov ratio gives the number of paired degrees of freedom in the near field and far field, the blurring of intensity XC prevents the determination of this number in the transition region. On the other hand, it can serve as a quantifier of the strength of blurring. On the contrary, the spectral number of degrees of freedom can be quantified through the XC Fedorov ratio at any *z*–axis position, as experimentally verified in Fig. 4(d). We remark that the evolution of spatial Fedorov ratio is very similar to that reported by^6^ in the single-photon regime.

Similarly, the ratio of beam width to AC width can be used to quantify the number of degrees of freedom of the individual down-converted beams constituting the twin beam^17^ both in space and spectrum and as functions of the *z*–axis position [see Fig. 4(c,d)]. The value of the AC ratio remains constant with propagation. This reflects the fact that once the ‘coherence structures’ of single beams are generated in the near field, they just propagate according to the diffraction theory that preserves the number of degrees of freedom. We note that no quantity analogous to the AC ratio exists at the single-photon level. We observe that the values of spectral AC ratio and XC Fedorov ratio shown in Fig. 4(d) differ by a multiplicative factor that has its origin in the detailed spectral structure of the measured twin beam^18^. We also note that the decrease in the values of spatial AC ratio observed in Fig. 4(c) close to the near field arises from the different shapes of the correlation functions in the near and far field^19^, whose contribution to the number of degrees of freedom is not precisely described by their FWHMs.

The actual number of degrees of freedom in the transverse plane can also be drawn from the experimental data using the heights of XC peaks measured over the whole transverse plane. All paired transverse modes contribute to multi-mode thermal statistics describing the field in this case, independent of the blurring of XC at different *z*–axis positions. The height of intensity XC peak and its comparison with the mean intensity reveals the number of modes, thus giving the number of degrees of freedom. The larger the number of degrees of freedom the weaker the intensity XC.

**Figure 5:**
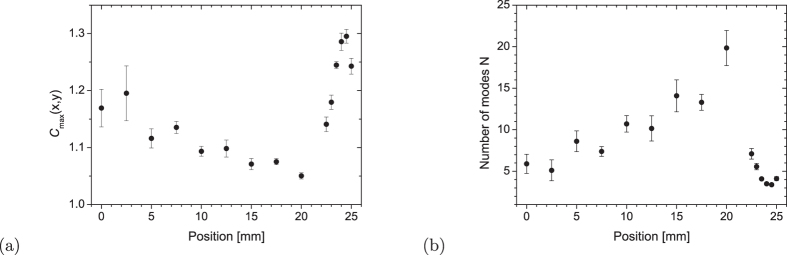
(**a**) Maximum height of the XC peak, *C*_*max*_, and (**b**) estimate for the number *N* of degrees of freedom of paired photons determined from the photon-number statistics as functions of the distance from the crystal; signal from 20 spectral pixels ×2 radial spatial pixels is added to give intensity.

The height of XC peak, *C*_*max*_, can also be used to monitor the blurring of XC, similarly as the Fedorov ratio, provided that the intensity XC is detected in a small area in the transverse plane. In this case, only a small number of degrees of freedom is observed in the near and far field due to the localization of the fields’ modes. At variance with this, practically all degrees of freedom contribute to this measurement for the *z*–axis positions where a large blurring of XC is observed. As a consequence, the number of detected degrees of freedom is nearly independent of the size of the detection area in this region. This contrasts with the behavior in the near and far field where the number of detected degrees of freedom increases linearly with the size of detection area. While the actual number of degrees of freedom in the whole twin beam cannot be obtained in our experimental setup, which does not monitor the whole transverse plane, the variation of *C*_*max*_ evaluated in a small area of the transverse plane has been observed [see Fig. 5(a)]. In the absence of noise, the value of *C*_*max*_ is related to the number *N* of degrees of freedom of paired photons measured by the detector by the formula *N* = 1/(*C*_*max*_ − 1)^16^,^20^,^21^. In the present case, the estimation of *N* is reliable since the amount of noise in the measurement is low due to the macroscopic nature of the twin beams. Figure 5(b) shows that, in the transition region, the number *N* of degrees of freedom dramatically increases. The result confirms that, even in a small collection area, a large number *N* of degrees of freedom contributes to the twin beam. These results can be compared to those obtained in^14^ in a different geometry.

## Discussion

Spatial and spectral coherence of high-intensity twin beams propagating from the near field to the far field has been experimentally studied by measuring intensity AC and XC functions, thus providing a complete characterization of the propagating twin beam. While the evolution of spatial and spectral intensity auto-correlation functions agrees with the usual evolution of the individual down-converted beams, the evolution of spatial intensity cross-correlation functions is crucially influenced by quantum correlations inside the twin beam. As a consequence, the near-field tight position cross correlations are completely blurred during the propagation and gradually replaced by tight momentum cross-correlations, ideally reached in the far-field. On the contrary, spectral intensity cross-correlations, similarly as the spectra of the down-converted beams, remain unchanged during the evolution. The propagation also preserves the number of spatial and spectral degrees of freedom (modes) constituting the twin beam. These numbers can be determined from the width of intensity AC peaks or, alternatively, from the height of intensity XC peaks measured over the whole transverse plane at any position of the propagating twin beam. Whereas the intensity XC functions in the high intensity regime behave qualitatively in the same way as those obtained in the single-photon regime, the intensity AC functions of intense twin beams naturally provide additional characterization of the beams.

## Methods

### Experimental setup

The experimental setup is depicted in Fig. 1. The third-harmonic beam from a picosecond-pulsed Nd:YLF laser (4.5 ps, 349 nm, 500 Hz) was used to pump a type-I BBO crystal (NLC, 4 mm long, cut Θ = 33.8 deg) in a nearly collinear configuration and placed on a roto-translation stage. In the beginning, the output face of the crystal was precisely set (distances *a*, *a*′ in Fig. 1) to be imaged to the input slit of an imaging spectrometer (Andor Shamrock 303i) by a lens with focal length *f* = 60 mm. The input slit was centered at the residual THG beam after most of the pumping energy was steered away with a bandwidth filter (BWF). After precise setting of distances *a* = 66 mm and *a*′ = 660 mm, the components were fixed to the optical table during the entire experiment. The chosen position corresponds to the near-field configuration with magnification *M* = *a*′/*a* = 10. During the experiment, the crystal was moved in the opposite direction with respect to the spectrometer, i.e. from the near-field position (at *z* = 24 mm in the sketch drawn in Section “Results”) up to the distance of 24 mm towards the far-field configuration (*z* = 0 mm in the sketch shown in Section “Results”). As we can see, few millimeters are enough to change the character of the spatial correlations from near-field-like to far-field-like. Since the pumping beam is well collimated, this translation along the z-axis does not significantly change the parameters of the interaction. The rotation stage is used to precisely set the opening angle of the PDC process so that the cone fits into the height of the input slit of the spectrometer.

The light from the PDC cone entering the input slit of the spectrometer gets dispersed at the grating (1200 rules per mm) in the horizontal plane. A portion of the spectrum between 683.4 nm and 712.5 nm can be registered at the iCCD detector (Andor iStar DH334T, 13 × 13 *μ*m^2^ pixel size). The spectral resolution of the system was 0.03 nm/pixel. The spectrum was centered approximately around the PDC degenerate wavelength (698 nm). A 50 *μ*m-wide slit was used throughout the experiment. The camera was operated at the maximum gain and maximum A/D conversion speed (5 MHz). The gating window of the camera was set to 5 ns, synchronous with the laser pulses, to ensure the detection of single shots of the PDC in each image. At each *z*-axis setting, a sequence of 10 thousands of camera frames (with full-resolution) was taken.

### Experimental determination of AC and XC functions

For a given *z*-axis position, the obtained sequences of images were processed in the following way. The *k* = 1…100 sample points 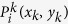 were taken in the right half of the *i*-th image (belonging to one of the down-converted beams), *x* and *y* being the spectral and spatial axes, respectively. For each point we computed the intensity correlation function


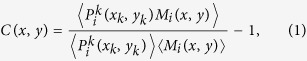


where *M*_*i*_(*x*, *y*) is the intensity profile of the *i*-th image of the sequence and 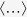 means averaging over the whole sequence. For each selected point 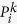 we got a correlation matrix that shows two peaks [see Fig. 2(c,d)]. One peak is centered around the selected point (*x*_*k*_, *y*_*k*_) in the area of the chosen down-converted beam. The other peak occurs at a conjugated point in the area of the second down-converted beam. A 2 × 2 software binning was performed to speed-up the processing without a significant loss of information due to the spatial and spectral resolution of the system. As a result, we get 100 processed images similar to the one shown in Fig. 2(c) for each sequence. In each of the processed images we identified the positions of the peaks and their widths (FWHM).

## Additional Information

**How to cite this article**: Haderka, O. *et al.* Spatial and spectral coherence in propagating high-intensity twin beams. *Sci. Rep.* **5**, 14365; doi: 10.1038/srep14365 (2015).
